# The Epidemiology of Newly Recognized Causes of Drug-Induced Liver Injury: An Update

**DOI:** 10.3390/ph17040520

**Published:** 2024-04-18

**Authors:** Einar Stefan Björnsson

**Affiliations:** Division of Gastroenterology and Hepatology, Department of Internal Medicine, The National University Hospital of Iceland, Faculty of Medicine, University of Iceland, Hringbraut, 101 Reykjavik, Iceland; einarsb@landspitali.is

**Keywords:** drug-induced liver injury, checkpoint inhibitors, COVID vaccination, AIH, DI-ALH, green tea extract

## Abstract

The incidence and prevalence of drug-induced liver injury appear to be increasing globally, for example, with the introduction of checkpoint inhibitors. Several reviews have been published in the last decade on the epidemiology of DILI, both among hospitalized patients and in the general population, as well as from retrospective and prospective studies on DILI. Most of these reviews have not focused on newly recognized agents that have recently changed the landscape of DILI. Apart from liver injury associated with antibiotics, oncological agents, particularly checkpoint inhibitors, are increasingly being recognized as causing liver injury. The type of liver injury associated with these agents is not *idiosyncratic* but rather an indirect type of injury. Furthermore, recently, COVID-19 vaccines and green tea extract have been found to lead to liver injury. Checkpoint inhibitors have revolutionized the treatment of many malignancies, such as malignant melanoma, lung cancer, and renal cancer. Via the activation of T cells, they can increase immune activity against malignant cells, but at the same time, they can decrease immune tolerance and therefore lead to immune-related adverse effects in many organs. The most common adverse effect in clinical practice is liver injury. A recent prospective study demonstrated an 8% frequency of DILI due to the use of checkpoint inhibitors among patients with malignant melanoma and renal cancer. This rate is much higher than observed with drugs, leading to *idiosyncratic* liver injury. Shortly after the implementation of the worldwide vaccination program against COVID-19, several case reports were published on suspected vaccination-induced autoimmune-like hepatitis occurring shortly after the vaccination. At first, these reports were met with skepticism, but currently, around 100 reports have been published, and cases of positive recurrence have been reported. The clinical, biochemical, immunological, and histological features are indistinguishable from classic autoimmune hepatitis (AIH). These reactions are very similar to drug-induced autoimmune-like hepatitis (DI-ALH) due to drugs such as nitrofurantoin, minocycline, and infliximab, which do not relapse after a short course of corticosteroids, which is the general rule in classic autoimmune hepatitis (AIH). Green tea extract has been found to be a well-documented cause of acute hepatocellular liver injury with jaundice. A strong HLA association has been reported, showing a high prevalence of HLA-B*35:01 among patients suffering from green tea-induced liver injury. Overall, 3% of patients recruited in the DILIN study were supplemented with green tea extract as one of the ingredients. In a prospective population-based study from Iceland, green tea was implicated in approximately 8% of patients with DILI.

## 1. Introduction

Drug-induced liver injury (DILI) is a potential adverse effect of many drugs, and idiosyncratic DILI is a major health concern [1]. DILI has attracted increasing interest among researchers in recent years, particularly with the introduction of new oncological agents, such as checkpoint inhibitors [2]. DILI is also a major concern for regulatory authorities that should ensure the safety of drugs to protect users and the pharmaceutical industry. DILI is one of the most common reasons for the termination of drug development of otherwise promising therapeutic agents in pre-clinical studies. DILI has become one of the major reasons for the withdrawal of drugs shortly after being put on the market. Furthermore, DILI is the most common cause of acute liver failure in many parts of the world. Thus, the overall burden of drug-induced liver injury seems to be increasing worldwide [1,2].

Apart from liver injury associated with antibiotics, which are among the most commonly used drugs in the world, oncological agents, particularly checkpoint inhibitors, are increasingly recognized as causes of liver injury. In a recently published paper from a reference hospital in Barcelona, anticancer drugs were the most common cause of DILI [1]. In the prospective European DILI network, recruiting patients from 2016 to 2021, the most common single causative drug classes were antibacterials (40%), followed by antineoplastic and/or immunomodulating agents (27%) [2]. In recent years, several papers have been published on the epidemiology of DILI [3,4,5,6]. The first studies on the epidemiology of DILI were retrospective studies that originated from the General Practitioners Database in the UK [7,8,9]. Only a few prospective studies on the incidence of DILI in the general population have been undertaken, such as those in France [10], Iceland [11], the US [12], and China [13] (Table 1). The first population-based study on DILI was undertaken in France during the years 1997–2000, reporting an annual crude incidence of 13.9 cases per 100,000 inhabitants per year [10]. If spontaneous reporting to the French Regulatory authorities was taken into consideration, DILI was at least 16 times more frequent than those obtained by spontaneous reporting [10]. Thus, if the results were extrapolated to the whole general population in France, more than 8000 cases could occur in France per year, leading to approximately 500 deaths [10]. The second population-based study performed was also a nationwide study that included the total population of Iceland and found an incidence of 19 cases per 100,000 inhabitants annually [11]. A much lower incidence of DILI was found in the first population-based study in the USA, with only 2.7 cases per 100,000 adults [12], than in previously mentioned studies from Europe [10,11]. The latest population-based study originated from Mainland China, reporting an estimated annual incidence in the general population of 23.8 per 100,000 persons [13]. The methodology of this study has been criticized [14]. The main criticism was based on the fact that there were no entrance criteria for diagnosis, especially not based on liver tests, the identity of drugs was not known in 44% of patients, and the study included predominantly hospitalized patients, which generally translates to cases of severe disease; thus, it is difficult to explain the low mortality of 0.39% [14]. Many studies have analyzed the proportion of patients with DILI among both hospitalized patients and outpatients [15,16,17,18,19,20,21,22]. Some retrospective studies have tried to assess the incidence of DILI. Crude incidence rates of DILI in the UK were reported to be 2.4 per 100,000 per year [17]. Remarkably, similar incidence rates of 2.3 per 100,000 were demonstrated among outpatients in a hepatology clinic in Sweden [21]. The incidence rates obtained in retrospective surveys are probably an underestimation of the true incidence due to the underreporting of adverse reactions, difficulties in finding these cases in medical record registries due to the lack of uniform diagnoses, and difficulties performing causality assessments in retrospective studies. Therefore, incidence figures obtained in prospective studies are probably more reliable as cases of DILI can be carefully searched for. In a study of DILI focused on an outpatient hepatology clinic in Sweden for the first time across a 10-year period, a DILI diagnosis was made in 6% of all cases, 3% were undergoing follow-up after hospitalization, and the other 3% of patients were referred liver test evaluations [21].

Recently, interest in hepatotoxicity related to the use of oncological drugs has increased considerably, and recommendations on the management of these patients are constantly changing as data on different phenotypes are emerging. A number of other agents have been reported in case reports to be associated with DILI, but the most commonly well-recognized implicated agents are checkpoint inhibitors.

Other agents that have recently been reported to lead to DILI and that have gained much interest recently are COVID-19 vaccines and herbal and dietary supplements (HDSs) that have green tea extract as one of the ingredients.

The aim of this review is to provide an update on the epidemiology and frequency of these relatively newly recognized agents, checkpoint inhibitors, COVID-19 vaccines, and HDSs that contain green tea extracts. Thus, DILI has been reported in relation to conventional drugs and HDSs.

## 2. Liver Injury Due to Checkpoint Inhibitors

The use of checkpoint inhibitors (CPIs) has revolutionized the treatment of many solid malignancies and has been shown to improve the prognosis of patients with advanced tumors, such as malignant melanoma and lung and renal cancer. Various types of checkpoint inhibitors have been developed, which are defined by the main targets of the CPIs, such as cytotoxic T lymphocyte-associated antigen-4 (CTLA4), programmed cell death-ligand-1 (PDL1), and programmed cell death protein-1 (PD1) [23]. Thus, via the activation of T cells, they can increase the immune activity against malignant cells. However, they can decrease immune tolerance, leading to adverse immune-related effects in many organs.

Liver injury associated with CPIs is a specific type of immune-mediated DILI and the so-called indirect type of DILI based on the immune-directed actions of the agents [24]. The cause of liver injury by agents leading to so-called indirect liver injury has more to do with what they do rather than what they are [24]. Indirect injury was recently identified as a separate entity from dose-dependent direct toxicity, such as that associated with an overdose of acetaminophen or idiosyncratic DILI [24]. A classic example is the induction of immune-mediated hepatitis due to TNF-alpha inhibitors or checkpoint inhibitors.

Studying the epidemiology of the hepatotoxicity associated with CPIs has been hampered by the definitions of liver injury, limited data on competing etiologies in clinical trials, cohorts originating from tertiary referral centers, and the differences between patients treated with monotherapy and combination therapies.

In clinical trials, ALT elevations have been reported in 3–15% of cases, with some being transient and others with 5–20 × ULN up to 3% [25,26,27,28]. Retrospective studies in tertiary referral centers have demonstrated that 2%-8% of patients had grade 3–4 ALT elevations [28,29,30,31].

However, data from clinical trials and retrospective studies could be an underestimation as real-life prospective studies have provided higher frequencies (see below). In a large cancer center in Texas, among 5762 patients treated with CPIs, 100 (2%) developed hepatotoxicity, occurring in a higher proportion of recipients of combination therapy (9.2%) when compared to monotherapy (up to 1.7%) [29]. Apart from liver injury associated with the use of CPIs, other immune-related adverse effects are common in these patients that can induce symptoms from many organs, such as colitis, pneumonitis, dermatitis, and hypophysitis. Hepatitis is the most common adverse effect encountered in clinical practice [29].

Patients receiving combination regimens have constantly been found to have a greater risk than those treated with monotherapy [29,30,31,32,33,34]. Previous studies have also shown that melanoma patients have a greater risk of hepatotoxicity as they often receive combination therapy in their treatment regimens. Several studies have found that CTLA-4 inhibitors have a higher risk of liver injury than anti-PD1 agents [29,30,31,32,33,34]. However, in a recent systematic review and network meta-analysis focused on monotherapy with CPIs, the overall risk of immune-mediated hepatotoxicity related to CTLA-4 inhibitors did not differ significantly from that of PD-1 inhibitors [34]. The overall incidence of hepatotoxicity was 4.1% [34]. The highest incidence of hepatotoxicity was observed with triple therapy, and the overall incidence of hepatotoxicity was similar between different dual regimens [34]. Interestingly, no direct relationship was found between the risk of liver injury and drug dose, whether monotherapy or combination therapy was used [34]. In another systematic review, a combination of CPIs was associated with a 5% rate of grade 3–4 [35], which was similar to the overall risk of 4.1% reported in a more recent review. However, retrospective real-life studies have demonstrated higher frequencies of hepatotoxicity. Among melanoma patients in the Netherlands treated with CPIs, severe hepatitis occurred in 20.7% associated with combination therapy of different CPIs, 2.6% for ipilimumab monotherapy, and 1.8% for PD-1 inhibitor [32]. In another study on melanoma patients from Canada, approximately half developed > grade 3 hepatotoxicity at a median of 34 days after the first dose [33]. Thus, real-life cohort studies have found higher frequencies of hepatotoxicity than what has been observed in clinical trials. However, causality assessments relating to DILI can be challenging in clinical practice in these patients, as has been demonstrated in recent studies undertaken by experts in DILI [36,37]. Patients treated with CPIs who have advanced malignancy can have both liver and bone metastases and often receive multiple drugs, and a thorough medical assessment is important to rule out competing etiologies.

A study from Michigan focused on patients receiving pembrolizumab for the treatment of solid organ tumors. In that study, only 20 (29%) liver injury cases were adjudicated as probable drug-induced DILI [36]. These patients were significantly more likely to have a hepatocellular/mixed injury pattern (65% vs. 12%) and be treated with corticosteroids (55% vs. 12%) and have lower mortality (45% vs. 76%) during follow-up [36].

In a landmark prospective study, all patients with melanoma and renal cancer treated with CPIs in a tertiary referral center at Nottingham University Hospital in the UK, as well as two other tertiary centers in Cambridge and Leeds from the same period, were included [37]. The patients underwent thorough medical evaluations, and causality assessments were performed with RUCAM [38], followed by an adjudication process carried out by experts in DILI. Hepatoxicity occurred in 38/432 (8.8%) of cases after the exclusion of 9/47 (19%) of patients with acute liver injury. The most commonly excluded patients were those who presented with cholestatic liver injury. The highest risk of liver injury was found in melanoma patients receiving a combination therapy of ipilimumab and nivolumab, followed by nivolumab, in approximately 28% [37]. In a study from Nottingham, the incidence rate was calculated using the number of patients and their time at risk and found an overall incidence of hepatotoxicity due to CPIs of 11.5 cases per 1000 person-months, and the highest incidence rate was observed among melanoma patients treated with combination therapy, in 38 per 1000 person-months [37]. The results of this prospective study demonstrated that despite the strict predetermined criteria, the frequency of DILI was higher than that reported in clinical trials [34,35]. The overall incidence rate of approximately 9% of DILI in this real-life study, which included a thorough causality assessment, is much higher than even the rates of the most frequent drugs that lead to idiosyncratic DILI. A prospective population-based study from Iceland, which took place prior to the marketing of CPIs, demonstrated that the highest risk of DILI was associated with users of azathioprine, occurring in 1 out of 133 users (0.8%) [12]. The second-highest risk was associated with infliximab, which is also a type of indirect liver injury, which was found in 1 out of 148 users (0.7%) [11]. Thus, it seems that the highest risk of hepatotoxicity reported so far is with the use of checkpoint inhibitors. The common use of CPIs has resulted in a high prevalence of DILI due to the use of these agents in recent DILI cohort studies [1,2,39]. After antibiotics, antineoplastic/immunomodulating agents were the second-most commonly implicated agents in the Pro-Euro-DILI (Prospective European DILI) study, being found in 27% of all DILI cases [2]. Remarkably, similar figures were reported from a prospective DILI registry in Australia, established in 2016, where antineoplastic/immunomodulating agents were the second-highest drug class after antibacterial drugs in terms of leading to DILI, as found in 22% of cases [39]. The aforementioned DILI registry studies from Europe and Australia started recruiting patients in 2016, which was before the widespread use of CPIs and, it was found that, in recent years, reports of liver injury seem to be increasing. In a recent study from Barcelona focused on referrals with suspicion of DILI from 2018 to 2023, the most common drug class leading to DILI were antineoplastic drugs in 20/76 (26%), most commonly nivolumab [1]. Apart from referrals to the hepatology unit, during the study period, an additional 126 patients with DILI due to CPIs who were not referred to a hepatologist were identified [1].

## 3. Liver Injury Due to COVID-19 Vaccines

Shortly after the implementation of the worldwide vaccination program against COVID-19, a case report by Bril et al. on suspected vaccination-induced autoimmune-like hepatitis was published [40]. These authors described a 35-year-old woman who developed jaundice one week after her first dose of the Pfizer-BioNTech vaccine. She was found to have experienced a hepatocellular reaction, with positive autoantibodies and a liver biopsy compatible with AIH. The authors raised concerns relating to vaccine-induced autoimmunity but were careful in their interpretation and suggested that the association could be coincidental [40]. However, during 2021 and 2022, at least 19 additional case reports or case series were published on this association [41,42,43,44,45,46,47,48,49,50,51,52,53,54,55,56,57,58,59,60,61,62]. Most of the patients reported have been isolated case reports; however, one series that included 16 patients has also been published [55]. Moreover, a study of this association from 18 countries, consisting mostly of previously published case reports, has been published [60], and a description of the histological and serological features of these patients has been analyzed [61]. The clinical features of the cases that developed autoimmune-like hepatitis shortly after the first report [42,43,44,45,46,47,48,49,50,51,52,53,54,55,56,57,58,59,60,61] were remarkably similar. Patients had a hepatocellular-type liver injury with classical biochemical features and histological and clinical features of genuine or classic autoimmune hepatitis. The vast majority of patients were treated with corticosteroids with a prompt response and the rapid resolution of their liver injury. As around 100 cases have been described, there does not seem to be any doubt that there is a causal relationship between vaccination and autoimmune-like hepatitis. In early reports, the authors themselves were hesitant to describe a causal relationship, and some have doubted causality due to the fact that liver test results have rarely been available prior to the administration of the vaccination [62]. However, a positive rechallenge has been reported [48]. Efe et al. described a liver injury occurring after the first dose of COVID-19 vaccine, with improvement of liver injury but a rapid increase in liver test values after the second dose was administered [60]. Thus, it seems clear that vaccination against COVID-19 can lead to “drug-induced autoimmune like hepatitis (DI-ALH). A report from a recent workshop on DI-ALH has been published in the Journal of Hepatology [63]. DI-ALH was defined as a liver injury with laboratory and/or histological features that are indistinguishable from autoimmune hepatitis (AIH). In the review, it was stated that more than 40 different drugs have been shown to have well-documented potential to cause DIöALH [63]. Drugs such as nitrofurantoin, hydralazine, methyldopa, minocycline, infliximab, and herbal and dietary supplements (such as Khat and Tinospora cordifolia) have been implicated in leading to DI-ALH. Unfortunately, specific markers of the disease that can distinguish between DI-ALH and genuine AIH are lacking. A management algorithm for patients with liver injury and an autoimmune phenotype was proposed in a review [63].

The conclusion of a workshop of international experts was that it is of great importance to differentiate DI-ALH from AIH, as patients with DI-ALH rarely require long-term immunosuppression, whereas patients with AIH mostly require long-term immunosuppression [63,64,65]. COVID-19-vaccination-induced autoimmune-like hepatitis is a type of DI-ALH. In around 100 reports, no AIH relapse has been reported [40,41,42,43,44,45,46,47,48,49,50,51,52,53,54,55,56,57,58,59,60,61,62]. This is similar to infliximab-induced AIH-like hepatitis [64] and other drugs with a well-documented capacity to induce autoimmune-like hepatitis [65]. A recent study on DI-ALH due to COVID-19 vaccination illustrated lobular hepatitis that was indistinguishable from the histological features of AIH and had similar serological features of autoimmunity [61]. In this study, there was no control group with AIH, but other studies have shown very similar biochemical, immunological, and histological features in patients with DI-ALH and AIH [64,65,66].

In a recent paper, it was correctly pointed out that no population-based studies have been undertaken on the risk and characteristics of liver injury following vaccination against COVID-19 [67]. The authors used a large database in Indiana and found unexplained liver test abnormalities in 0.038% of individuals following SARS-CoV-19 vaccination [67]. However, a major limitation of the study acknowledged by the authors is that manual chart review was not undertaken to adjudicate liver injury in order to determine the causal relationship between vaccine and liver injury. As has been pointed out, database studies cannot identify DILI without a causality assessment [68].

In conclusion, the COVID-19-vaccination-induced autoimmune hepatitis-like phenotype has been well documented in around 100 patients, and there seems no doubt that there is a causal relationship, as seen with other drugs, which leads to DI-ALH. AIH-like hepatitis has also been reported after other vaccinations [69,70]. As with other drugs that lead to DI-ALH, this type of liver injury is important to recognize due to the fact that long-term immunosuppression is not required in contrast to patients with genuine or classic AIH that is not related to the use of drugs.

## 4. Liver Injury from Green Tea Extract

Herbal and dietary supplements (HDSs) are popular in many countries, with one study showing that 30–40% of the adult US population regularly used HDSs [71]. Thus, in clinical practice, it is of great importance to ask if patients are taking HDSs as a part of their medical history.

Green tea derived from the leaves of the Camellia sinensis plants has been considered to have beneficial effects on health and has been marketed to increase energy levels and general well-being but mostly for weight loss, although data to support the beneficial effects are largely lacking.

Liver reactions have been well documented for several HDSs. In DILI studies from Asia and India, liver injury associated with HDSs is very common [18,19,22]. Thus, HDSs are well recognized causes of liver injury. In many cases, causality assessments can be difficult in patients with liver injury after the intake of HDSs due to the lack of information on the potential hepatotoxicity of the sometimes multiple ingredients. Liver injury due to HDSs has been increasingly recognized in Europe and North America in recent decades.

In the Spanish Hepatotoxicity Liver Injury registry, the proportion of cases attributed to HDSs was only 2% in 2006 [72] but increased to 13% for the period 2010–2013 [73]. Similarly, in a prospective DILIN study, HDSs accounted for 16% of those diagnosed with liver injury related to both conventional drugs and HDSs [74]. This increased from 7% during 2004–2005 to 19% in 2010–2012 and they are now thought to be involved in around 20% of cases [75]. 

The incidence of HDS-associated liver injury in a prospective population-based study from Iceland was found to be involved in 15/96 (16%) of cases, which were found to be attributed to the use of HDSs [12]. The overall crude incidence was 19 per 100,000, and the year and incidence of HDS-related acute liver injury was 3 per 100,000 persons [12]. In Table 2, the proportion of HDS among patients with DILI is illustrated as well as those with HDS with green tea extract included.

Liver injury associated with the use of green tea extract has been increasingly reported in the medical literature [79,80,81,82,83,84].

In the original case reports of suspected liver injury due to green tea extract, the biochemical phenotype was remarkably similar to severe acute hepatocellular reactions, often with prolonged jaundice [80,81,82,83]. Although these original reports were sometimes met with skepticism, cases with positive rechallenge were reported [79,80], supporting the role of green tea extract in causing liver injury.

In a landmark paper from the DILIN study, 40/1414 (3%) of the patients enrolled were attributed to the use of HDSs with green tea as one of the components [85]. As in previous reports, the liver injury was hepatocellular in 95%, and 83% of patients presented with jaundice [76]. Latency from the intake of green tea extract occurred within 1 to 3 months of starting to use the product. In most of the cases, the liver injury was self-limiting. However, the course was judged as severe in 14 patients (35%), necessitating liver transplantation in three (8%) [85]. In three cases, a positive rechallenge was reported. Interestingly, HLA typing revealed a high prevalence of HLA-B*35:01, which was found in 72% of green tea cases compared to only 15% caused by other supplements, which was similar to the 11% rate seen in the population controls [85].

As with other HDSs that lead to liver injury, the risk associated with their use in terms of hepatotoxicity is unclear. In contrast to information on the use of conventional drugs that can be made available by authorities, at least in some countries [11], data relating to the sale and use of HDSs are usually unavailable. Thus, the frequency of green tea-induced liver injury is unclear in the general population. As mentioned above, 3% of cases with well-documented liver injury were found in the DILIN study to be related to the use of supplements that contain green tea extract. However, the DILIN study is not population-based. As mentioned above, in a population-based study from Iceland, 16% (*n* = 15) of cases were attributed to the use of HDSs [11]. Among HDSs, 8/15 (53%) of cases with HDS-associated liver injury used HDSs that included green tea extract, including the following HDSs: Mega men heart^®^, Metasys^®^, serious mass^®^, and different Herbalife products (*n* = 5) [12]. Thus, compared with the 3% rate found in the DILIN study, overall, eight out of ninety-six (8.3%) had green tea-induced liver injury in an Icelandic study [12]. In a Latin America DILI study, among forty cases with liver injury due to HDS, eight (18%) were associated with supplements that included green tea extract [20].

To conclude, liver injury associated with the use of checkpoint inhibitors, COVID-19 vaccines, and green tea extract has been well established. DILI associated with checkpoint inhibitors seems to be more frequent in real-life studies than idiosyncratic liver injury. COVID-19 vaccines can induce drug-induced autoimmune hepatitis-like hepatitis, which responds well to corticosteroids, and patients have not been reported to experience a relapse of liver injury after initial recovery. Green tea extract has a distinct clinical, biochemical, and histological phenotype with hepatocellular injury with a strong HLA risk factor.

## Figures and Tables

**Table 1:** The table shows prospective studies performed on DILI, showing the study period, crude incidence rate per 100,000 inhabitants annually, gender proportions, most common drug type, and mortality. Ref = reference.

	Sgro et al., 2002 Ref. [10] *n* = 34	Bjornsson et al., 2013 Ref. [11] *n* = 96	Vega et al., 2017 Ref. [12] *n* = 23	Shen et al., 2019 Ref. [13] *n* = 25,927
Country	France	Iceland	USA	China
Study period	1997–2000	2010–2011	2014	2012–2014
Incidence	13.9 per 100,000	19.1 per 100,000	2.7 per 100,000	23.8 per 100,000
Females (%)	65%	54%	57%	49%
Most common drug type	Antibiotics	Antibiotics	Antibiotics	HDS
Mortality	2/34 (5.9%)	1/96 (1%)	0%	0.39%

**Table 2:** The table shows the most frequently implicated drugs and the proportion of herbal dietary supplements (HDSs) in large prospective DILI registries. The proportion with HDS-related injury due to green tea extract in HDS-implicated liver injury. Ref = reference; AAS = anabolic steroids.

	Bjornsson et al., 2013 Ref. [11] *n* = 96	Chalasani et al., 2015 Ref. [76] *n* = 899	Stephens et al., 2017 Ref. [77] *n* = 843	Devarbhavi et al., 2017 Ref. [78] *n* = 1288	Bjornsson et al., 2023 Ref. [2] *n* = 246
Country	Iceland	USA	Spain	India	Europe
Study period	2010–2011	2004–2013	1994–2018	2013–2018	2016–2021
Most common drug	Amoxicillin–clavulanate	Amoxicillin–clavulanate	Amoxicillin–clavulanate	Anti-TBC drugs	Amoxicillin–clavulanate
HDS and AAS	16%	16%	6%	14%	6%
Green tea extract (%)	8/96 (8.3%)	40/1440 (4%)	-	-	-

## Data Availability

Data sharing is not applicable.

## References

[1] Pocurull A., Moreta M.J., Heitman D., Olivas I., Collazos C., Canga E., Sáez-Peñataro J., Andrade R.J., Lucena M.I., Mariño Z. (2024). Anticancer drugs are the first cause of drug-induced liver injury in a reference hospital. Liver Int..

[2] Björnsson E.S., Stephens C., Atallah E., Robles-Diaz M., Alvarez-Alvarez I., Gerbes A., Weber S., Stirnimann G., Kullak-Ublick G., Cortez-Pinto H. (2023). A new framework for advancing in drug-induced liver injury research. The Prospective European DILI Registry. Liver Int..

[3] Ahmad J., Odin J.A. (2017). Epidemiology and Genetic Risk Factors of Drug Hepatotoxicity. Clin. Liver Dis..

[4] Bjornsson E.S. (2019). Global epidemiology of drug-induced liver injury (DILI). Curr. Hepatol. Rep..

[5] Bjornsson E.S. (2020). Epidemiology, predisposing factors and outcome in DILI. Clin. Liver Dis..

[6] Li X., Tang J., Mao Y. (2022). Incidence and risk factors of drug-induced liver injury. Liver Int..

[7] Jick H., Derby L.E., García Rodríguez L.A., Jick S.S., Dean A.D. (1992). Liver disease associated with diclofenac, naproxen, and piroxicam. Pharmacotherapy.

[8] García Rodríguez L.A., Williams R., Derby L.E., Dean A.D., Jick H. (1994). Acute liver injury associated with nonsteroidal anti-inflammatory drugs and the role of risk factors. Arch. Intern. Med..

[9] Rodríguez L.A.G., Stricker B.H., Zimmerman H.J. (1996). Risk of acute liver injury associated with the combination of amoxicillin and clavulanic acid. Arch. Intern. Med..

[10] Sgro C., Clinard F., Ouazir K., Chanay H., Allard C., Guilleminet C., Lenoir C., Lemoine A., Hillon P. (2002). Incidence of drug-induced hepatic injuries: A French population-based study. Hepatology.

[11] Bjornsson E.S., Bergmann O.M., Bjornsson H.K., Kvaran R.B., Olafsson S. (2013). Incidence, Presentation and Outcomes in Patients with Drug-Induced Liver Injury in the General Population of Iceland. Gastroenterology.

[12] Vega M., Verma M., Beswick D., Bey S., Hossack J., Merriman N., Shah A., Navarro V. (2017). The incidence of drug- and herbal and dietary supplement- induced liver injury: Preliminary findings from gastroenterologist-based surveillance in the population of the state of Delaware. Drug Saf..

[13] Shen T., Liu Y., Shang J., Xie Q., Li J., Yan M., Xu J., Niu J., Liu J., Watkins P.B. (2019). Incidence and Etiology of Drug-Induced Liver Injury in Mainland China. Gastroenterology.

[14] Devarbhavi H., Bjornsson E.S. (2019). Letter to the Editor: Response to the article “Incidence and Etiology of Drug-Induced Liver Injury in Mainland China”. Gastroenterology.

[15] Bagheri H., Michel F., Lapeyre-Mestre M., Lagier E., Cambus J.P., Valdiguié P., Montastruc J.L. (2000). Detection and incidence of drug-induced liver injuries in hospital: A prospective analysis from laboratory signals. Br. J. Clin. Pharmacol..

[16] Meier Y., Cavallaro M., Roos M., Pauli-Magnus C., Folkers G., Meier P.J., Fattinger K. (2005). Incidence of drug-induced liver injury in medical inpatients. Eur. J. Clin. Pharmacol..

[17] de Abajo F.J., Montero D., Madurga M., Garcia Rodriguez L.A. (2004). Acute and clinically relevant drug-induced liver injury: A population based case-control study. Br. J. Clin. Pharmacol..

[18] Suk K.T., Kim D.J., Kim C.H., Park S.H., Yoon J.H., Kim Y.S., Baik G.H., Kim J.B., Kweon Y.O., Kim B.I. (2012). A prospective nationwide study of drug-induced liver injury in Korea. Am. J. Gastroenterol..

[19] Wai C.-T., Tan B.-H., Chan C.-L., Sutedja D.-S., Lee Y.-M., Khor C., Lim S.-G. (2007). Drug-induced liver injury at an Asian center: A prospective study. Liver Int..

[20] Bessone F., García-Cortés M., Medina-Caliz I., Hernandez N., Parana R., Mendizabal M., Schinoni M.I., Ridruejo E., Nunes V., Peralta M. (2021). Herbal and dietary supplements-induced liver injury in Latin America: Experience from the LATINDILI network. Clin. Gastroenterol. Hepatol..

[21] de Valle M., Klinteberg V., Alem N., Olsson R., Björnsson E. (2006). Drug-induced liver injury in a Swedish University hospital outpatient hepatology clinic. Aliment. Pharmacol. Ther..

[22] Devarbhavi H., Dierkhising R., Kremers W.K., Sandeep M.S., Karanth D., Adarsh C.K. (2010). Single-center experience with drug-induced liver injury from India: Causes, outcome, prognosis and predictors of mortality. Am. J. Gastroenterol..

[23] Larkin J., Chiarion-Sileni V., Gonzalez R., Grob J.-J., Cowey C.L., Lao C.D., Schadendorf D., Dummer R., Smylie M., Rutkowski P. (2015). Combined nivolumab and ipilimumab or monotherapy in untreated melanoma. N. Engl. J. Med..

[24] Hoofnagle J.H., Bjornsson E.S. (2019). Drug Induced Liver Injury: Types and Phenotypes. N. Eng. J. Med..

[25] Sznol M., Ferrucci P.F., Hogg D., Atkins M.B., Wolter P., Guidoboni M., Lebbé C., Kirkwood J.M., Schachter J., Daniels G.A. (2017). Pooled analysis safety profile of nivolumab and ipilimumab combination therapy in patients with advanced melanoma. J. Clin. Oncol..

[26] Wolchok J.D., Chiarion-Sileni V., Gonzalez R., Rutkowski P., Grob J.-J., Cowey C.L., Lao C.D., Wagstaff J., Schadendorf D., Ferrucci P.F. (2017). Overall survival with combined nivolumab and ipilimumab in advanced melanoma. N. Engl. J. Med..

[27] Suzman D.L., Pelosof L., Rosenberg A., Avigan M.I. (2018). Hepatotoxicity of immune checkpoint inhibitors: An evolving picture of risk associated with a vital class of immunotherapy agents. Liver Int..

[28] De Martin E., Michot J.M., Papouin B., Champiat S., Mateus C., Lambotte O., Roche B., Antonini T.M., Coilly A., Laghouati S. (2018). Characterization of liver injury induced by cancer immunotherapy using immune checkpoint inhibitors. J. Hepatol..

[29] Miller E.D., Abu-Sbeih H., Styskel B., Nogueras Gonzalez G.M., Blechacz B., Naing A., Chalasani N. (2020). Clinical Characteristics and Adverse Impact of Hepatotoxicity due to Immune Checkpoint Inhibitors. Am. J. Gastroenterol..

[30] Gauci M.L., Baroudjian B., Bédérède U., Zeboulon C., Delyon J., Allayous C., Madelaine I., Eftekhari P., Resche-Rigon M., Poté N. (2021). Severe immune-related hepatitis induced by immune checkpoint inhibitors: Clinical features and management proposal. Clin. Res. Hepatol. Gastroenterol..

[31] Malnick S.D.H., Abdullah A., Neuman M.G. (2021). Checkpoint inhibitors and hepatotoxicity. Biomedicines.

[32] Biewenga M., van der Kooij M.K., Wouters M.W., Aarts M.J., van den Berkmortel F.W., de Groot J.W.B., Boers-Sonderen M.J., Hospers G.A.P., Piersma D., van Rijn R.S. (2021). Checkpoint inhibitor induced hepatitis and the relation with liver metastasis and outcome in advanced melanoma patients. Hepatol. Int..

[33] Smith M.K., Chan Y., Suo A.E., Shaheen A.A., Congly S.E., Tandon P., Bhanji R.A., Wells M.M., Cheng T., Ma C. (2021). Clinical Course and Treatment Implications of Combination Immune Checkpoint Inhibitor-Mediated Hepatitis: A Multicentre Cohort. J. Can. Assoc. Gastroenterol..

[34] Zheng C., Huang S., Lin M., Hong B., Ni R., Dai H., Lin X., Yang J. (2023). Hepatotoxicity of immune checkpoint inhibitors: What is Currently Known. Hepatol. Commun..

[35] Arnaud-Coffin P., Maillet D., Gan H.K., Stelmes J.J., You B., Dalle S., Péron J. (2019). A systematic review of adverse events in randomized trials assessing immune checkpoint inhibitors. Int. J. Cancer.

[36] Tsung I., Dolan R., Lao C.D., Fecher L., Riggenbach K., Yeboah-Korang A., Fontana R.J. (2019). Liver injury is most commonly due to hepatic metastases rather than drug hepatotoxicity during pembrolizumab immunotherapy. Aliment. Pharmacol. Ther..

[37] Atallah E., Welsh S.J., O’Carrigan B., Oshaughnessy A., Dolapo I., Kerr A.S. (2023). Incidence, risk factors and outcomes of checkpoint inhibitor-induced liver injury: A 10-year real-world retrospective cohort study. JHEP Rep..

[38] Danan G., Benichou C. (1993). Causality assessment of adverse reactions to drugs-I. A novel method based on the conclusions of international consensus meetings: Application to drug-induced liver injuries. J. Clin. Epidemiol..

[39] Trinh S., Batt N., Sawhney R., Nicoll A. (2023). The importance of prospective drug-induced liver injury registries. Liver Int..

[40] Bril F., Al Diffalha S., Dean M., Fettig D.M. (2021). Autoimmune hepatitis developing after coronavirus disease 2019 (COVID-19) vaccine: Causality or casualty?. J. Hepatol..

[41] Vuille-Lessard É., Montani M., Bosch J., Semmo N. (2021). Autoimmune hepatitis triggered by SARS-CoV-2 vaccination. J. Autoimmun..

[42] Garrido I., Lopes S., Simões M.S., Liberal R., Lopes J., Carneiro F., Macedo G. (2021). Autoimmune hepatitis after COVID-19 vaccine—More than a coincidence. J. Autoimmun..

[43] Rocco A., Sgamato C., Compare D., Nardone G. (2021). Autoimmune hepatitis following SARS-CoV-2 vaccine: May not be a casuality. J. Hepatol..

[44] Capecchi P.L., Lazzerini P.E., Brillanti S. (2021). Comment on “Autoimmune hepatitis developing after coronavirus disease 2019 (COVID-19) Vaccine: Causality or casualty?”. J. Hepatol..

[45] Avci E., Abasiyanik F. (2021). Autoimmune hepatitis after SARSCoV-2 vaccine: New-onset or flare-up?. J. Autoimmun..

[46] Londoño M.C., Gratacós-Ginès J., Sáez-Peñataro J. (2021). Another case of autoimmune hepatitis after SARS-CoV-2 vaccination—Still casualty?. J. Hepatol..

[47] Clayton-Chubb D., Schneider D., Freeman E., Kemp W., Roberts S.K. (2021). Autoimmune hepatitis developing after the ChAdOx1 nCoV-19 (Oxford-AstraZeneca) vaccine. J. Hepatol..

[48] Tan C.K., Wong Y.J., Wang L.M., Ang T.L., Kumar R. (2021). Autoimmune hepatitis following COVID-19 vaccination: True causality or mere association?. J. Hepatol..

[49] Kang S.H., Kim M.Y., Cho M.Y., Baik S.K. (2022). Autoimmune Hepatitis Following Vaccination for SARS-CoV-2 in Korea: Coincidence or Autoimmunity?. J. Korean Med. Sci..

[50] Lodato F., Larocca A., D’Errico A., Cennamo V. (2021). An unusual case of acute cholestatic hepatitis after m-RNABNT162b2 (Comirnaty) SARS-CoV-2 vaccine: Coincidence, autoimmunity or drug-related liver injury. J. Hepatol..

[51] Ghielmetti M., Schaufelberger H.D., Mieli-Vergani G., Cerny A., Dayer E., Vergani D., Terziroli Beretta-Piccoli B. (2021). Acute autoimmune-like hepatitis with atypical anti-mitochondrial antibody after mRNA COVID-19 vaccination: A novel clinical entity?. J. Autoimmun..

[52] McShane C., Rigby C.K.J., Crosbie Ó. (2021). The mRNA COVID-19 vaccine—A rare trigger of autoimmune hepatitis?. J. Hepatol..

[53] Mahalingham A., Duckworth A., Griffiths W.J.H. (2022). First report of post-transplant autoimmune hepatitis recurrence following SARS-CoV-2 mRNA vaccination. Transpl. Immunol..

[54] Boettler T., Csernalabics B., Salié H., Luxenburger H., Wischer L., Salimi Alizei E., Zoldan K., Krimmel L., Bronsert P., Schwabenland M. (2022). SARS-CoV-2 vaccination can elicit a CD8 T-cell dominant hepatitis. J. Hepatol..

[55] Shroff H., Satapathy S.K., Crawford J.M., Todd N.J., VanWagner L.B. (2022). Liver injury following SARS-CoV-2 vaccination: A multicenter case series. J. Hepatol..

[56] Palla P., Vergadis C., Sakellariou S., Androutsakos T. (2022). Letter to the editor: Autoimmune hepatitis after COVID-19 vaccination: A rare adverse effect?. Hepatology.

[57] Zin Tun G.S., Gleeson D., Al-Joudeh A., Dube A. (2022). Immune mediated hepatitis with the Moderna vaccine, no longer a coincidence but confirmed. J. Hepatol..

[58] Cao Z., Gui H., Sheng Z., Xin H., Xie Q. (2022). Letter to the editor: Exacerbation of autoimmune hepatitis after COVID-19 vaccination. Hepatology.

[59] Suzuki Y., Kakisaka K., Takikawa Y. (2022). Letter to the editor: Autoimmune hepatitis after COVID-19 vaccination: Need for population-based epidemiological study. Hepatology.

[60] Efe C., Kulkarni A.V., Terziroli Beretta-Piccoli B., Magro B., Stättermayer A., Cengiz M., Clayton-Chubb D., Lammert C., Bernsmeier C., Gül Ö. (2022). Liver injury after SARS-CoV-2 vaccination: Features of immune-mediated hepatitis, role of corticosteroid therapy and outcome. Hepatology.

[61] Codoni G., Kirchner T., Engel B., Villamil A.M., Efe C., Stättermayer A.F., Weltzsch J.P., Sebode M., Bernsmeier C., Lleo A. (2022). Histological and serological features of acute liver injury after SARS-CoV-2 vaccination. JHEP Rep..

[62] Mungmunpuntipantip R., Wiwanitkit V. (2021). Lettter to the editor on “Autoimmune hepatitis after COVID-19 vaccination”. Hepatology.

[63] Andrade R.J., Aithal G.P., De Boer Y.S., Liberal R., Gerbes A., Regev A., Beretta-Piccoli B.T., Schramm C., Kleiner D.E., De Martin E. (2023). Nomenclature, Diagnosis and Management of Drug-induced Autoimmune-like hepatitis (DI-ALH): An expert opinion meeting report. J. Hepatol..

[64] Björnsson H.K., Gudbjörnsson B., Björnsson E.S. (2021). Infliximab-induced liver injury: Clinical phenotypes, autoimmunity and the role of corticosteroid treatment. J. Hepatol..

[65] Björnsson E., Talwalkar J., Treeprasertsuk S., Kamath P.S., Takahashi N., Sanderson S., Neuhauser M., Lindor K. (2010). Drug-induced autoimmune hepatitis: Clinical characteristics and prognosis. Hepatology.

[66] Suzuki A., Brunt E.M., Kleiner D.E., Miquel R., Smyrk T.C., Andrade R.J., Lucena M.I., Castiella A., Lindor K., Björnsson E. (2011). The use of liver biopsy evaluation in discrimination of idiopathic autoimmune hepatitis versus drug-induced liver injury. Hepatology.

[67] Guardiola J., Lammert C., Teal E., Chalasani N. (2022). Unexplained liver test elevations after SARS-CoV-2 vaccination. J. Hepatol..

[68] Björnsson H.K., Björnsson E.S. (2021). Database Studies on Drug-Induced Liver Injury: The Importance of Causality Assessment. Am. J. Gastroenterol..

[69] Sasaki T., Suzuki Y., Ishida K., Kakisaka K., Abe H., Sugai T., Takikawa Y. (2018). Autoimmune hepatitis following influenza virus vaccination: Two case reports. Medicine.

[70] Van Gemeren M.A.J., van Wijngaarden P., Doukas M., de Man R.A. (2017). Vaccine-related autoimmune hepatitis: The same disease as idiopathic autoimmune hepatitis? Two clinical reports and review. Scand. J. Gastroenterol..

[71] Bailey R.L., Gahche J.J., Lentino C.V., Dwyer J.T., Engel J.S., Thomas P.R., Betz J.M., Sempos C.T., Picciano M.F. (2011). Dietary supplement use in the United States, 2003–2006. J. Nutr..

[72] Andrade R.J., Lucena M.I., Fernández M.C., Palaez G., Pachkoria K., Garcia-Ruiz E., García-Muñoz B., González-Grande R., Pizarro A., Durán J.A. (2005). Spanish Group for the Study of Drug-Induced liver Disease. Drug-induced liver injury: An analysis of 461 incidences submitted to the Spanish Registry over a 10-year period. Gastroenterology.

[73] Robles-Diaz M., Gonzalez-Jimenez A., Medina-Caliz I., Stephens C., Garcia-Cortes M., GarciaMunoz B., Ortega-Alonso A., Blanco-Reina E., Gonzalez-Grande R., Jimenez-Perez M. (2015). Distinct phenotype of hepatotoxicity associated with illicit use of anabolic androgenic steroids. Aliment. Pharmacol. Ther..

[74] Navarro V.J., Barnhart H., Bonkovsky H.L., Davern T., Fontana R.J., Grant L., Reddy K.R., Seeff L.B., Serrano J., Sherker A.H. (2014). Liver injury from herbals and dietary supplements in the U.S. Drug-Induced Liver Injury Network. Hepatology.

[75] Navarro V., Khan I., Björnsson E., Seef L.B., Serrano J., Hoofnagle J.H. (2017). Liver Injury from Herbal and Dietary Supplements. Hepatology.

[76] Chalasani N., Bonkovsky H.L., Fontana R.J., Lee W., Stolz A., Talwalkar J., Reddy K.R., Watkins P.B., Navarro V., Barnhart H. (2015). Features and out- comes of 899 patients with drug-induced liver injury: The DILIN prospective study. Gastroenterology.

[77] Stephens C., Roblez-Diaz M., Medina-Caliz I., Garcia-Cortes M., Ortega-Alonso A., Sanabria-Cabrera J., Gonzalez-Jimenez A., Alvarez-Alvarez I., Slim M., Jimenez-Perez M. (2021). Comprehensive analysis and insights gained from long-term experience of the Spanish DILI Registry. J. Hepatol..

[78] Devarbhavi H., Tarun J., Kumar N.S., Rathi C., Thomas V., Singh S.P., Sawant P., Goel A., Eapen C.E., Rai P. (2021). The Indian network of drug- induced liver injury: Etiology, clinical features, outcome and prog- nostic markers in 1288 patients. J. Clin. Exp. Hepatol..

[79] Bonkovsky H.L. (2006). Hepatotoxicity associated with supplements containing Chinese green tea (*Camella sinensis*). Ann. Intern. Med..

[80] Jimenez-Saenz M., del Carmen Martinez-Sanchez M. (2006). Acute hepatitis associated with the use of green tea infusions. J. Hepatol..

[81] Björnsson E., Olsson R. (2007). Serious adverse liver reactions associated with herbal weight loss supplements. J. Hepatol..

[82] Dara L., Hewett J., Lim J.K. (2008). Hydroxycut hepatotoxicity: A case series and review of liver toxicity from herbal weight loss supplements. World J. Gastroenterol..

[83] Navarro V.J., Bonkovsky H.L., Hwang S.I., Vega M., Barnhart H., Serrano J. (2013). Catechins in dietary supplements and hepatotoxicity. Dig. Dis. Sci..

[84] Mazzanti G., Di Sotto A., Vitalone A. (2015). Hepatotoxicity of green tea: An update. Arch. Toxicol..

[85] Hoofnagle J.H., Bonkovsky H.L., Phillips E.J., Li Y.J., Ahmad J., Barnhart H., Durazo F., Fontana R.J., Gu J., Khan I. (2021). HLA-B*35:01 and Green Tea-Induced Liver Injury. Drug-Induced Liver Injury Network. Hepatology.

